# The binding of an anti-PD-1 antibody to FcγRΙ has a profound impact on its biological functions

**DOI:** 10.1007/s00262-018-2160-x

**Published:** 2018-04-23

**Authors:** Tong Zhang, Xiaomin Song, Lanlan Xu, Jie Ma, Yanjuan Zhang, Wenfeng Gong, Yilu Zhang, Xiaosui Zhou, Zuobai Wang, Yali Wang, Yingdi Shi, Huichen Bai, Ning Liu, Xiaolong Yang, Xinxin Cui, Yanping Cao, Qi Liu, Jing Song, Yucheng Li, Zhiyu Tang, Mingming Guo, Lai Wang, Kang Li

**Affiliations:** BeiGene (Beijing) Co., Ltd., No. 30 Science Park Road, Zhong-Guan-Cun Life Science Park, Changping District, Beijing, 102206 People’s Republic of China

**Keywords:** PD-1, Antibody, FcγRI, Macrophages, Cancer therapy

## Abstract

Antibodies targeting PD-1 have been demonstrated durable anti-cancer activity in certain cancer types. However, the anti-PD-1 antibodies are less or not efficacious in many situations, which might be attributed to co-expression of multiple inhibitory receptors or presence of immunosuppressive cells in the tumor microenvironment. Most of the anti-PD-1 antibodies used in clinical studies are of IgG4 isotype with the S228P mutation (IgG4_S228P_). The functional impact by the interaction of anti-PD-1 IgG4_S228P_ antibody with Fc gamma receptors (FcγRs) is poorly understood. To assess the effects, we generated a pair of anti-PD-1 antibodies: BGB-A317/IgG4_S228P_ and BGB-A317/IgG4-variant (abbreviated as BGB-A317), with the same variable regions but two different IgG4 Fc-hinge sequences. There was no significant difference between these two antibodies in binding to PD-1. However, BGB-A317/IgG4_S228P_ binds to human FcγRI with high affinity and mediates crosslinking between PD-1 and FcγRI. In contrast, BGB-A317 does neither. Further cell-based assays showed that such crosslinking could reverse the function of an anti-PD-1 antibody from blocking to activating. More importantly, the crosslinking induces FcγRI^+^ macrophages to phagocytose PD-1^+^ T cells. In a mouse model transplanted with allogeneic human cancer cells and PBMCs, BGB-A317 showed significant tumor growth inhibition, whereas BGB-A317/IgG4_S228P_ had no such inhibition. Immunohistochemistry study revealed an inverse correlation between FcγRI^+^ murine macrophage infiltration and the density of CD8^+^PD-1^+^ human T cells within tumors in the BGB-A317/IgG4_S228P_-treated group. These evidences suggested that FcγRI^+^ binding and crosslinking had negative impact on the anti-PD-1 antibody-mediated anti-cancer activity.

**Electronic supplementary material:**

The online version of this article (10.1007/s00262-018-2160-x) contains supplementary material, which is available to authorized users.

## Introduction

Immune surveillance plays a critical role in cancer prevention. However, in situations where tumors develop resistance mechanisms to suppress the host immune system, tumors eventually grow out of control [1, 2]. One of such resistance mechanism is the up-regulation of the immune check-point ligand, PD-L1, in tumor cells or in tumor-associated immune cells. PD-L1 interacts with PD-1 (programmed cell death-1) on T cells, inhibiting T-cell proliferation and effector functions such as cytokine secretion and tumor cell-killing [3, 4]. Several PD-1 antagonist antibodies have been tested in clinical trials, and show significant efficacy in the treatment of advanced cancer types [5–9]. Two anti-PD-1 antibodies, nivolumab, and pembrolizumab recently gained regulatory approval [3].

Antibody drugs exert primary pharmacodynamic effects through specific binding to a target protein and modulating its functional activity via the variable regions. Furthermore, the constant region of an antibody also plays important roles by exerting secondary pharmacodynamic effects through the binding to FcγRs or activation of complement cascade. Each IgG subclass has a unique set of features for binding to effector receptors that elicit profound functional effects on the target cells [10, 11].

Most of the anti-PD-1 monoclonal antibodies (mAb), including nivolumab and pembrolizumab, have IgG4_S228P_ heavy chain, which retains effector-binding functions similar to that of wild-type human IgG4 [11], while it possesses more stable dimeric structure without fab-arm exchange observed in wild-type IgG4 [12, 13]. It was well documented that human IgG4 has significant binding to high affinity FcγRI through the Fc-hinge regions [11]. IgG4_S228P_ antibodies likely retain the binding to FcγRI. In a syngeneic mouse model, an anti-PD-1 mAb with “effector-less” Fc region demonstrated superior anti-tumor efficacy as compared with the one with effector functions [14]. However, functional consequences of FcγRI engagement by anti-PD-1 mAb through IgG4_S228P_ have not been well studied.

FcγRI is highly expressed in type 2 macrophages (M2) under inflammatory conditions in certain tumor types [15]. It is also expressed in myeloid-derived suppressor cells (MDSCs) and type I macrophages (M1). The functions induced by FcγRI engagement span a wide scope of cellular activities including antibody-dependent cell phagocytosis (ADCP), cell proliferation, and production of cytokines depending on the cell type in which FcγRI is activated [16].

In this report, we studied the functional consequences of an anti-PD-1 mAb with an IgG4_S228P_ heavy chain, for which we generated a pair of anti-PD-1 antibodies with the same variable regions, but with different forms of the IgG4 heavy chain: BGB-A317/IgG4_S228P_ (with a single S228P mutation) and BGB-A317 (lack of FcγR-binding capacity). Comparative characterization of these two mAbs demonstrated that BGB-A317/IgG4_S228P_ binds to human FcγRI with high affinity and mediates crosslinking between PD-1^+^ T cells and FcγRI^+^ cells. In addition, the two BGB-A317 mAbs showed profound differences in their capacity to modulate T-cell and macrophage functions in vitro or inhibit tumor growth using xenograft model in vivo.

## Materials and methods

### Binding affinity assay by SPR

For the characterization of the binding affinity of BGB-A317 or BGB-A317/IgG4_S228P_ to human PD-1, the extracellular domain of the human PD-1 protein, with a His tag (PD-1/His), was coupled to an activated CM5 biosensor chip (Biacore^®^, GE Healthcare Life Sci). BGB-A317 or BGB-A317/IgG4_S228P_ samples were injected and binding responses to human PD-1/His were calculated by subtracting the response unit (RU) from the values measured for a blank flow cell. Association rates (*K*_on_) and dissociation rates (*K*_off_) were calculated using the BIA Evaluation Software (GE Life Sciences). The equilibrium dissociation constant (*K*_D_) was calculated as the ratio of *K*_off_/*K*_on_, and binding affinity was calculated as *K*_on_/*K*_off_.

For the analysis of the binding of BGB-A317 and BGB-A317/IgG4_S228P_ to human FcγRI, human PD-1/His was immobilized on a CM5 chip. FcγRI was injected over a human PD-1/His-captured antibody surface. The sandwich binding of PD-1, BGB-A317/IgG4_S228P_, and FcγRI was performed by flowing FcγRI protein on top of the PD-1, BGB-A317/IgG4_S228P_ complex bound on the sensor chip. The same procedure was applied to BGB-A317.

### Generation of stable cell lines

A Chimeric PD-1 receptor, P3Z, was constructed by fusing the extracellular and transmembrane domains of human PD-1 to the cytoplasmic domain of human CD3ζ chain, which was stably transduced into HuT78 cells (ATCC) to generate HuT78/P3Z cells. The HEK293/PD-L1 and HEK293/FcγRI cell lines were generated by stable transfection. HuT78/P3Z cells were cultured in complete RPMI1640 (Hyclone) supplemented with 10% heat-inactivated FBS (Corning), and incubated at 37 °C with 5% CO_2_. HEK293-based cell lines were cultured in DMEM (Invitrogen) supplemented with 10% FBS (Corning).

### P3Z assays

HuT78/P3Z cells were pre-incubated with BGB-A317 (0.0014-3 µg/mL) for 15 min prior to co-culturing with HEK293/PD-L1 cells in 96-well plates (Costar) containing complete RPMI1640 media. The cells were incubated for 17 h at 37 °C. IL-2 secretion in the supernatants of co-culture was assayed by ELISA using a kit from eBioscience, according to manufacturer’s instructions.

### Human PBMC separation

Human peripheral blood mononuclear cells (PBMCs) were isolated from healthy donors by density gradient centrifugation using Histopaque-1077 (Sigma) according to the manufacturer’s instructions. All procedures were approved by the Internal Review Board at BeiGene. Consent agreement forms were signed before blood donation.

### ELISA

In the ELISA-based assay, bait proteins (1 µg/mL, 100 ng/well) were coated onto MaxiSorp plates (Thermo Fisher). For BGB-A317 antibody binding to PD-1, the bait protein is PD-1/his. For assessing antibody and FcγR binding, the extracellular domain of FcγR, e.g., FcγRI/his, was coated in the wells. A preformed immune-complex mixture was added to wells and incubated at room temperature for 1–2 h. The preforming immune-complex reaction contained 60 ng/mL streptavidin-HRP, 60 ng/mL of biotinylated F(ab′)2 goat anti-human IgG (F(ab′)2) (Jackson ImmunoRes, USA), and 1 µg/mL of BGB-A317 or BGB-A317/IgG4_S228P_ or human IgG (huIgG) in the blocking buffer. ELISA-binding signals were detected by Immobilon chemiluminescence substrate A/B (Millipore).

### Generation of M2 macrophages

Generation of M2 macrophages was performed according to the protocol described by Leidi et al. [15]. Briefly, human PBMCs were co-cultured in 6-well plates or 100-mm culture dish (Corning) in complete RPMI1640 media supplemented with 30 ng/ml human M-CSF (R&D systems) for 4 days. Adherent cells were retained by gently washing off non- and loose-adherent cells, with half of media replaced, and culture for 2–3 more days. For M2 polarization, 10 ng/ml IL-10 (Peprotech) was added during the last 48 h of culture.

### ADCP

HuT78/PD-1 cells were labeled with carboxyfluorescein succinimidyl ester (CFSE) (Life technologies) according to the manufacturer’s instructions. M2 macrophages were detached by Accutase™ and plated in V-bottom 96-well plates with CFSE-labeled HuT78/PD-1 at a ratio of 2:1 (HuT78/PD-1 cell to macrophage) in the presence of PD-1 mAbs for 5 h at 37 °C. After co-culturing, cells were stained with anti-CD11b-APC and subjected to flow cytometry. The percentage of macrophages that underwent ADCP of HuT78/PD-1 was determined by FACS of double positive cells (CFSE^+^ and CD11b^+^) following gating on CD11b^+^ M2 macrophages.

### FACS analysis

FACS analysis was performed using Guava^®^ easyCyte 6HT or 8HT (Millipore Merck). For the cell-based-binding assay, FcγRI-transfected HEK293 cells (HEK293/FcγRI) were stained with BGB-A317 or BGB-A317/IgG4_S228P_ or huIgG, followed by detection with AlexaFluor 488-conjugated goat F(ab′)2 anti-human IgG (F(ab′)2) fragment (Jackson ImmunoRes). The cell surface binding signals were quantified as mean fluorescence intensities (MFIs).

For the analysis of tumor infiltrated immune cells from the mouse in vivo cancer models, tumor tissue was cut into small pieces and digested with collagenase type I (1 mg/ml, Sigma) and 100 µg/ml DNase I (Sigma) in RPMI1640 plus 5% FBS for 30 min at 37 °C. Single cell suspension was obtained after passing the digested tissues through a 70 µm cell strainer. The cells were then washed and blocked by human IgG, followed by staining with human CD3 (HIT3a, 4A biotech), CD8 (OKT8, Sungene biotech), PD-1 (MIH4, eBioscience), CD64 (10.1, eBioscience), or mouse CD64 (X54-5/7.1, Biolegend) antibodies at 4 °C. The stained samples were subjected to flow cytometry and analysis using guavaSoft3.1.1 (Millipore, Merck).

### In vivo efficacy study

NOD/SCID mice (purchased from Vital River) were pre-treated with cyclophosphamide (150 mg/kg, J&K) intraperitoneally (i.p.) once a day for 2 days. One day after the second dose, animals were injected subcutaneously (s.c.) with 2.5 × 10^6^ A431 cells (ATCC) and 5 × 10^6^ PBMCs (a total of 200 µl cell mixture in 50% matrigel) in the right front flank. Starting from day 0 after cell inoculation, animals were randomly grouped and then treated as indicated. Primary tumor volume was measured twice every week, using a caliper. All experiments were conducted based on the protocols approved by the Animal Care and Use Committee of BeiGene according to the guidelines of the Chinese Association for Laboratory Animal Sciences.

### Pharmacokinetics analysis of BGB-A317 and BGB-A317/IgG4_S228P_

Mouse blood samples were collected from retro-orbital sinus at indicated time points. The concentration of BGB-A317 or BGB-A317/IgG4_S228P_ in serum was determined by ELISA. Briefly, serum samples were added to PD-1/His protein (2 µg/ml)-coated ELISA plates (Nunc), followed by the capture with anti-huIgG-HRP (Sigma) and color development. OD values at 450 nm were detected by a Microplate Reader (SpectraMax^®^ Paradigm^®^). The results were analyzed with SoftMax Pro software (Molecular Devices).

### IHC and immunofluoresence staining

Tumor tissues were harvested and fixed in formalin, dehydrated, embedded in paraffin, sectioned at 3 µm, and placed on polylysine-coated slides. The sections were deparaffinized in xylene and rehydrated in graded ethanol. Antigen retrieval was performed in citrate buffer (pH 6.0) by boiling for 30 min in a microwave and cooling down to room temperature. Then, the sections were blocked by 3% bovine serum albumin in PBS for 1 h and 0.3% H_2_O_2_ solution in PBS for 10 min, and afterwards, stained by the antibodies against human CD8 (SP16, ZSGB-Bio), PD-1 (NAT105, Abcam), CD64 (3D3, Abcam), and mouse CD64 (Clone 027, Sino Biological) at 4 °C overnight. The antibodies were detected by HRP conjugated second antibodies and DAB. The immunofluorescence staining was performed using Opal™ 4 or 7-color immunofluorescence staining kits (PerkinElmer). The images were acquired on the Vectra System and were analyzed using inForm software (both from PerkinElmer).

### Statistical analysis

Student’s *t* test was used to analyze differences between groups. *P* < 0.05 was considered statistically significant. Statistical analysis was done by GraphPad Prism software (GraphPad, La Jolla, CA).

## Results

### Generation of anti-PD-1 antibodies with different binding properties to FcγRs

The humanized anti-PD-1 mAb, hu317-4b6 (published in the patent US8735553 B1), binds to human PD-1 with high affinity and blocks PD-1 ligand (either PD-L1 or PD-L2)-mediated signaling (data not shown). To investigate the functionality of anti-PD-1 IgG4 antibodies with or without Fc-mediated effector functions, the variable region of hu317 was linked to two variant forms of human IgG4 constant regions, respectively, resulting in two different antibodies. One, named BGB-A317 (being tested in clinical trial, ClinicalTrials.gov number: NCT02407990), is incapable of binding to FcγRs due to several mutations in its Fc-hinge region. The other antibody, referred to as BGB-A317/IgG4_S228P_, is identical to the wild-type IgG4 except S228P mutation. The majority of anti-PD-1 antibodies (including nivolumab and pembrolizumab) are of the IgG4_S228P_ format. The differences of three IgG4 formats, including wild type, S228P and that of BGB-A317, are shown by sequence alignment of the Fc-hinge regions (Suppl. Figure 1).

**Fig. 1:**
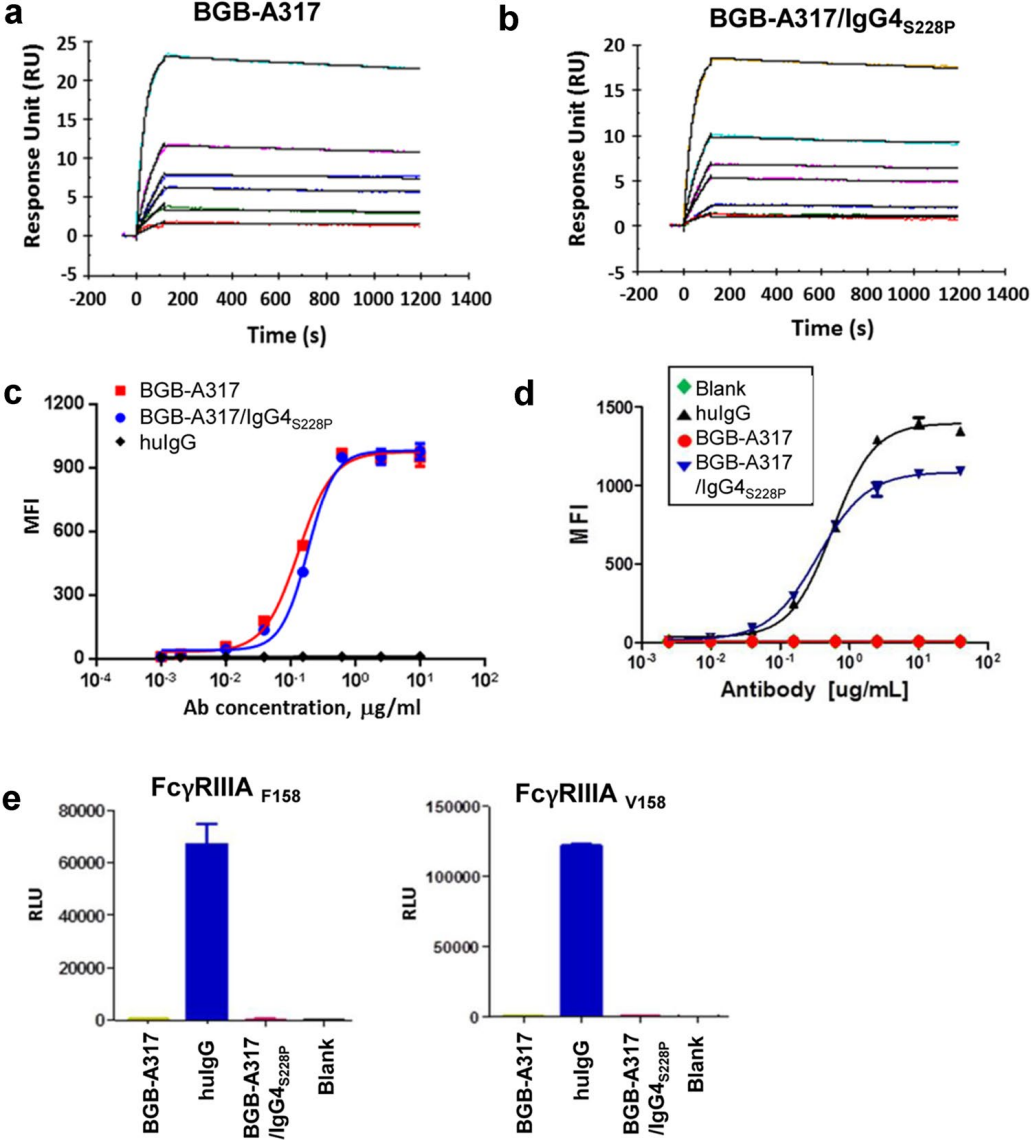
Comparison of BGB-A317 and BGB-A317/IgG4_S228P_ binding profiles to human PD-1, FcγRI, and FcγRIIIA. **a, b** Real-time SPR sensorgrams of BGB-A317 and BGB-A317/IgG4_S228P_ binding to human PD-1 assayed using BIAcore. *Y*-axis, response unit (RU). *X*-axis, reaction time course, seconds. **c** Binding of BGB-A317 and BGB-A317/IgG4_S228P_ to HuT78/PD-1 cells assayed by FACS. Mean fluorescence intensity (MFI) was determined by flow cytometry. **d** Differential binding of BGB-A317 and BGB-A317/IgG4_S228P_ to FcγRI assayed by FACS using HEK293/FcγRI cells. **e** Both BGB-A317 and BGB-A317/IgG4_S228P_ have low binding activity for FcγRIIIA, determined by ELISA. The anti-PD-1 antibodies were incubated with secondary antibodies to form immune complexes before adding to FcγRIIIA-coated 96-well plates. Bound immune complexes were detected with a chemiluminescence substrate. HuIgG was used as a positive control

Binding assays using SPR technology demonstrated that both BGB-A317 and BGB-A317/IgG4_S228P_ bind to PD-1 equally well (Fig. 1a, b). FACS analysis of antibody binding to surface PD-1 showed similar results (Fig. 1c). However, the two antibodies have very different binding affinity to certain FcγRs, most notably, FcγRI (high affinity FcγR). BGB-A317/IgG4_S228P_ binds to FcγRI with high affinity (*K*_*A*_) and low dissociation constant (*K*_*D*_) (Fig. 1d and Suppl. Table 1), and it also has significant binding to FcγRIIA and FcγRIIB (data not shown). In contrast, BGB-A317 has no binding to FcγRI (Fig. 1d) as well as to other FcγRs. The FcγR-binding profiles of BGB-A317/IgG4_S228P_ are very similar to other IgG4 antibodies such as nivolumab and pembrolizumab (data not shown), as well as those reported for regular human IgG4 [10, 17]. BGB-A317/IgG4_S228P_ exhibits low binding to FcγRIIIA (Fig. 1e), in consistent with other IgG4_S228P_ antibodies.

### BGB-A317/IgG4_S228P_ but not BGB-A317 mediates the crosslinking between PD-1 and FcγRI

**Fig. 2:**
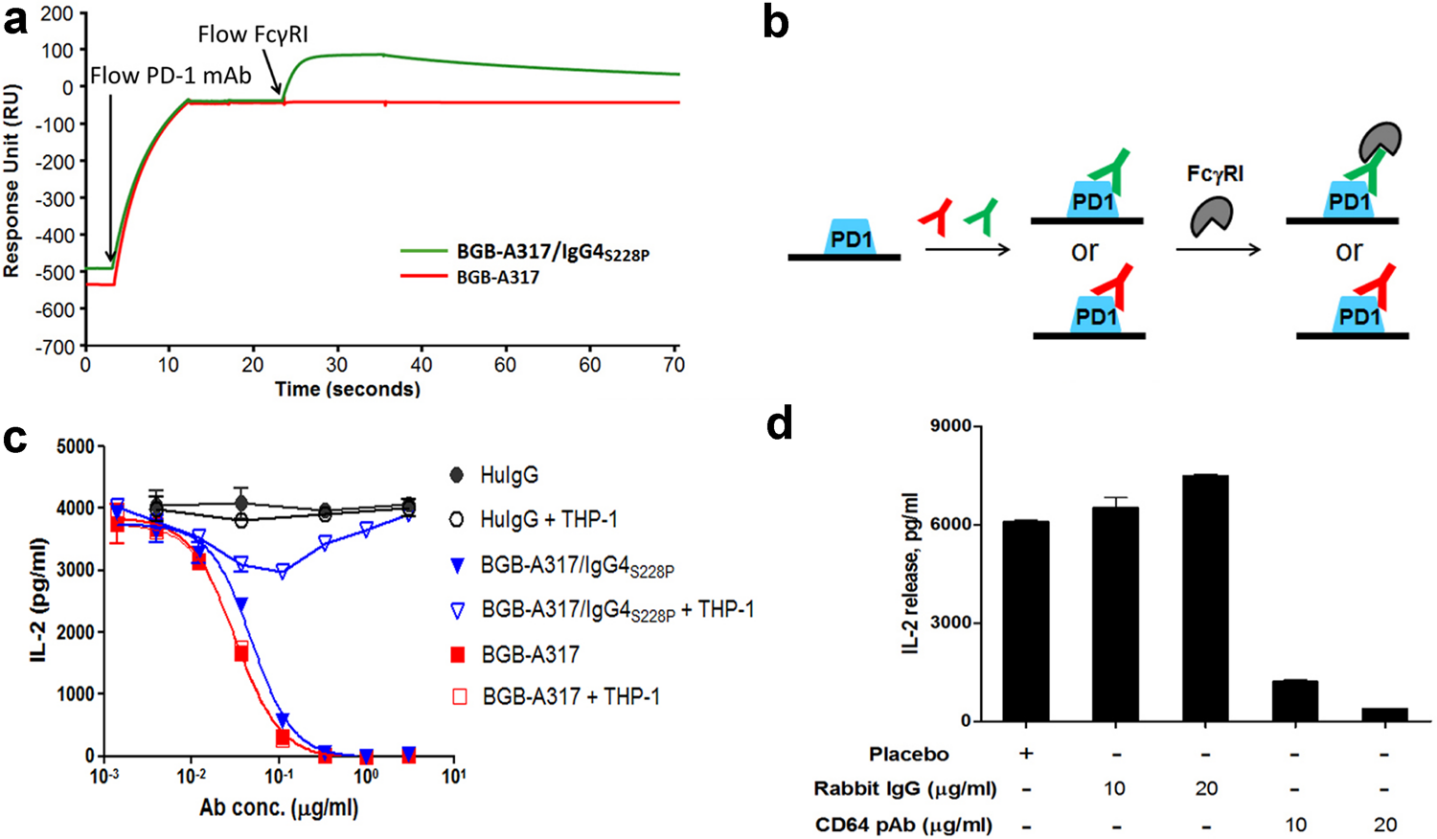
BGB-A317/IgG4_S228P_ mediates crosslinking between PD-1 and FcγRI receptors determined by biochemical and cell signaling assays. **a** Overlaid sensorgrams of BGB-A317 and BGB-A317/IgG4_S228P_ binding to PD-1 followed by binding with FcγRI. The PD-1 antibody (either BGB-A317 or BGB-A317/IgG4_S228P_) was injected onto the surface of human PD-1/His-coated CM5 chip, followed by FcγRI association and dissociation. The arrows indicated the time points when the analytes were injected. **b** Cartoon interpretation of the SPR assay in (**a**) showing that BGB-A317/IgG4_S228P_ mediates crosslinking between PD-1 and FcγRI, while BGB-A317 does not. **c** Comparison of PD-1 antibody functional activities by P3Z assay. In the assays, detection of IL-2 secretion served as a quantitative indicator of PD-1 signaling. The dose–response was assayed either in the “two-cell line” co-cultures (HuT78/P3Z and HEK293/PD-L1) presented by solid symbols or in the “three-cell line” system (HuT78/P3Z, HEK293/PD-L1 and FcγRI^+^ THP-1 cells) presented by open symbols. *Ab conc*. Antibody concentration. **d** Rabbit Anti-CD64 (FcγRIα) polyclonal antibody (pAb) restored the inhibitory effect of BGB-A317/IgG4_S228P_ on PD-1-signaling in the “three-cell” co-culture system. THP-1 cells were pre-treated with the rabbit Anti-CD64 pAb before co-culturing with HuT78/P3Z and HEK293/PD-L1 cells in the presence of BGB-A317/IgG4_S228P_. The placebo contained antibody buffer solution only

The functions of an antibody are not only determined by its binding to the target protein but also impacted by Fc-mediated effector functions. Since BGB-A317/IgG4_S228P_ has significant binding to FcγRI, we next investigated whether effective crosslinking between PD-1 and FcγRI could be established by BGB-A317/IgG4_S228P_ using SPR. The sensorgram readout clearly demonstrated that only BGB-A317/IgG4_S228P_ forms stable crosslinking between PD-1 and FcγRI although both BGB-A317 and BGB-A317/IgG4_S228P_ could bind to chip-bound PD-1 (Fig. 2a), which is explained by Fig. 2b.

The functional consequences of the crosslinking were tested by reverse signaling assay in cell co-culture system with or without the addition of FcγR-expressing myeloid cells (THP-1). Thus, PD-1 signaling upon crosslinking is manifested by T-cell activation. In the reverse signaling assay using two-cell co-culture system including the signal sensor cell, HuT78/P3Z, that expresses the chimeric PD-1 receptor (see “Materials and methods”) and the signal sending cell, HEK293/PD-L1, The engagement of chimeric receptor P3Z with PD-L1 leads to activating, instead of inhibiting the sensor cell to secrete IL-2. In this system, both BGB-A317 and BGB-A317/IgG4_S228P_ blocked PD-1-mediated signaling equally well (Fig. 2c). However, the addition of a third cell line, THP-1 (FcγRI^+^), to the above two-cell co-culture system resulted in dramatic changes in BGB-A317/IgG4_S228P_ function: inhibition of IL-2 secretion was reversed as the PD-1 antibody concentration increased (Fig. 2c), indicating that the crosslinking functions to activate PD-1-mediated signaling, rather than to block the T-cell signaling in the sensor cells. In contrast, BGB-A317 function was not affected by the presence of FcγRI^+^ THP-1 in the co-culture assay (Fig. 2c).

To further determine the role of FcγRI in the crosslinking of PD-1 receptor, rabbit anti-CD64 (FcγRI α subunit, or FcγRIα) polyclonal antibodies were added to the system. As shown in Fig. 2d, BGB-A317/IgG4_S228P_-mediated crosslinking was indeed reversed in the presence of anti-CD64 neutralizing antibody, suggesting that FcγRI plays a major role in this process. These observations are consistent with previous findings that crosslinking can change an inhibitory antibody into an agonist antibody [18, 19].

### BGB-A317/IgG4_S228P_ induces macrophage phagocytosis of PD-1^+^ T cells and *IL-10* gene expression

**Fig. 3:**
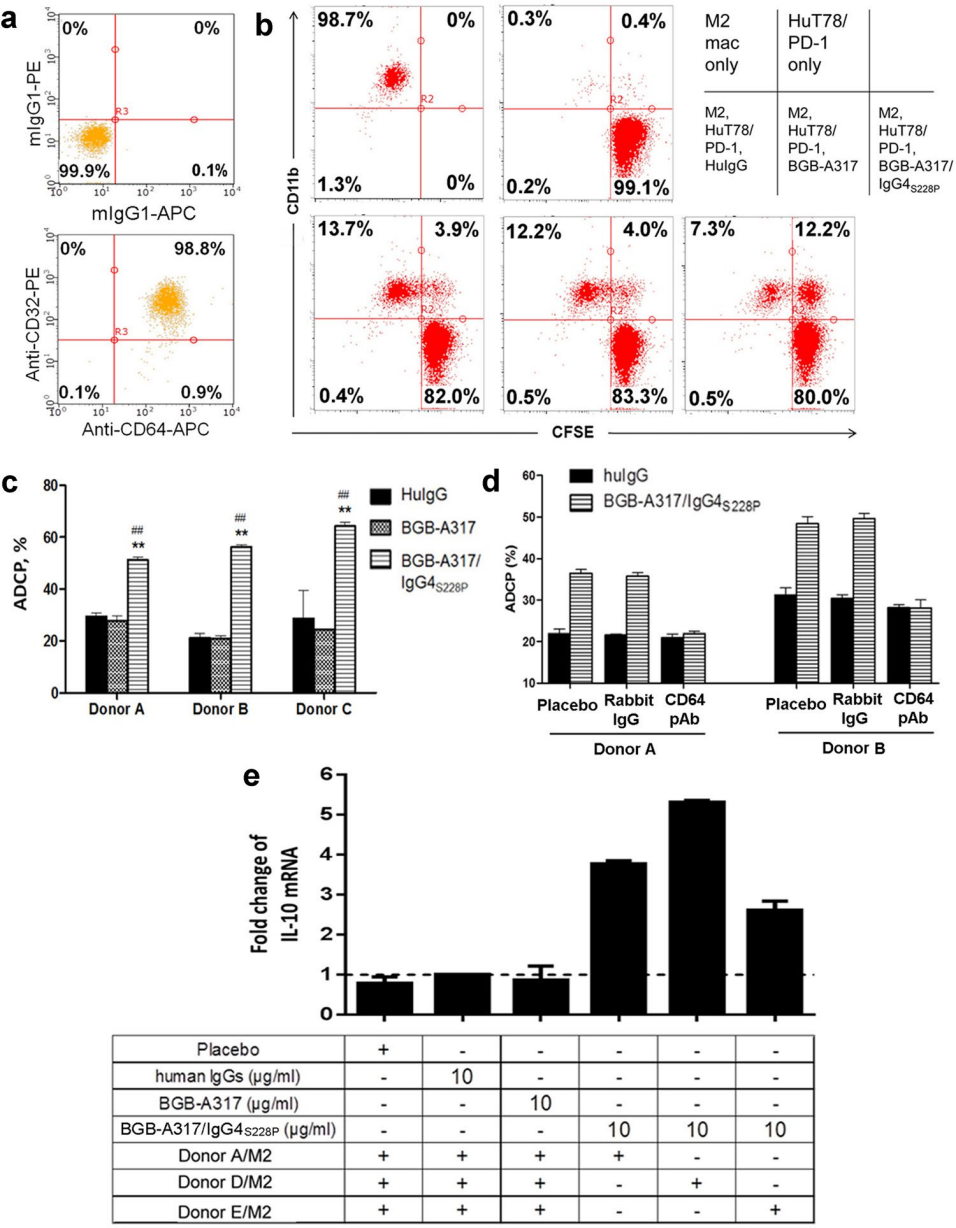
BGB-A317/IgG4_S228P_ induces primary M2 macrophages to phagocytose PD-1^+^ T cells (ADCP) and to express *IL-10* gene. **a** CD64 (FcγRI) and CD32 (FcγRII) expression on the in vitro differentiated M2 macrophages as determined by FACS. **b** ADCP assay using M2 macrophages. Primary M2 macrophages were co-cultured with CFSE-labeled PD-1^+^ HuT78/PD-1 cells overnight in the presence of the indicated antibodies. HuIgG was used as negative control. Representative dot plots of three independent experiments are shown. M2 Mac: M2 macrophage. **c** Bar graphs summarized the results of three independent experiments. The % of ADCP was determined as described in the “Materials and methods”. The mean + SD represents triplicate data points. ^##^*P* < 0.01, comparing BGB-A317/IgG4_S228P_ versus huIgG. **P < 0.01, comparing BGB-A317/IgG4_S228P_ versus BGB-A317. **d** Rabbit anti-CD64 polyclonal antibody neutralizes FcγRI-mediated ADCP. Rabbit IgG and placebo were used as negative controls. **e** Crosslinking of FcγRI^+^ M2 macrophages to plate-coated PD-1 by BGB-A317/IgG4_S228P_ induces *IL-10* gene expression. M2 macrophages were added to PD-1-coated 96-well plates in the presence of anti-PD-1 Abs and cultured overnight. The *IL-10* gene expression was assayed by real-time PCR. *IL-10* expression in huIgG-treated M2 macrophages was set as a baseline. The mRNA levels of placebo (antibody buffer solution), BGB-A317 or BGB-A317/IgG4_S228P_-treated M2 macrophages were normalized against the baseline. The results from three independent experiments are shown as mean + SD of duplicate data points. HuIgG is a mixture of human IgG1, IgG2, IgG3 and IgG4 (Invitrogen)

PBMC-derived type 2 macrophages (M2) express high levels of FcγRI (CD64) and FcγRII (CD32) (Fig. 3a). Therefore, it is very likely that anti-PD-1 antibody with IgG4_S228P_ Fc may trigger FcγRI-mediated signaling in macrophages upon binding to PD-1. To test this hypothesis, we investigated whether BGB-A317/IgG4_S228P_ or BGB-A317 could induce macrophage phagocytosis of PD-1^+^ T cells (antibody-dependent cell phagocytosis, ADCP). Co-culture of M2 macrophages with HuT78/PD-1 cells in the presence of BGB-A317/IgG4_S228P_ resulted in a significant increase of ADCP. In contrast, treatment with BGB-A317 only had baseline ADCP readouts (Fig. 3b, c). Furthermore, rabbit polyclonal antibody against CD64 could almost completely block BGB-A317/IgG4_S228P_-mediated ADCP (Fig. 3d). These results provided the compelling evidence that a PD-1 antibody with FcγRI-binding activity could induce ADCP via crosslinking of PD-1^+^ T cells and FcγRI^+^ macrophages, and FcγRII seemed not to play any significant role.

Several studies showed that engagement of FcγRI not only induced ADCP, but also activated the gene transcription of anti-inflammatory cytokine IL-10 in macrophages and promoted M2 macrophage generation [19, 20]. Therefore, we monitored *IL-10* gene expression in M2 macrophages after crosslinking of FcγRI. The assay was performed by seeding M2 macrophages in PD-1-coated plates in the presence of BGB-A317/IgG4_S228P_ or BGB-A317. Quantitative RT-PCR analysis demonstrated that *IL-10* gene transcription was up-regulated by up to fivefold, when BGB-A317/IgG4_S228P_ was added to the cell culture (Fig. 3e). Neither BGB-A317 nor negative control antibodies affect *IL-10* gene transcription. The results indicated that the anti-PD-1 IgG4_S228P_ antibody could engage FcγRI^+^ macrophages, induce ADCP and activate transcription of *IL-10* gene via crosslinking. Furthermore, we determined the cytokine production in the co-culture of M2 macrophages with HuT78/PD-1 cells in the presence of anti-PD-1 antibodies. The results showed that significantly higher amounts of macrophage cytokines (IL-10, IL-8, IL-6 and TNF-α) [21] were produced after treatment with anti-PD-1/IgG4_S228P_ antibodies than that with BGB-A317 and control huIgG despite of some cross-individual variations (Suppl. Figure 3).

### BGB-A317 and BGB-A317/IgG4_S228P_ elicited significantly different anti-tumor activities in a xenograft allogenic cancer model

**Fig. 4:**
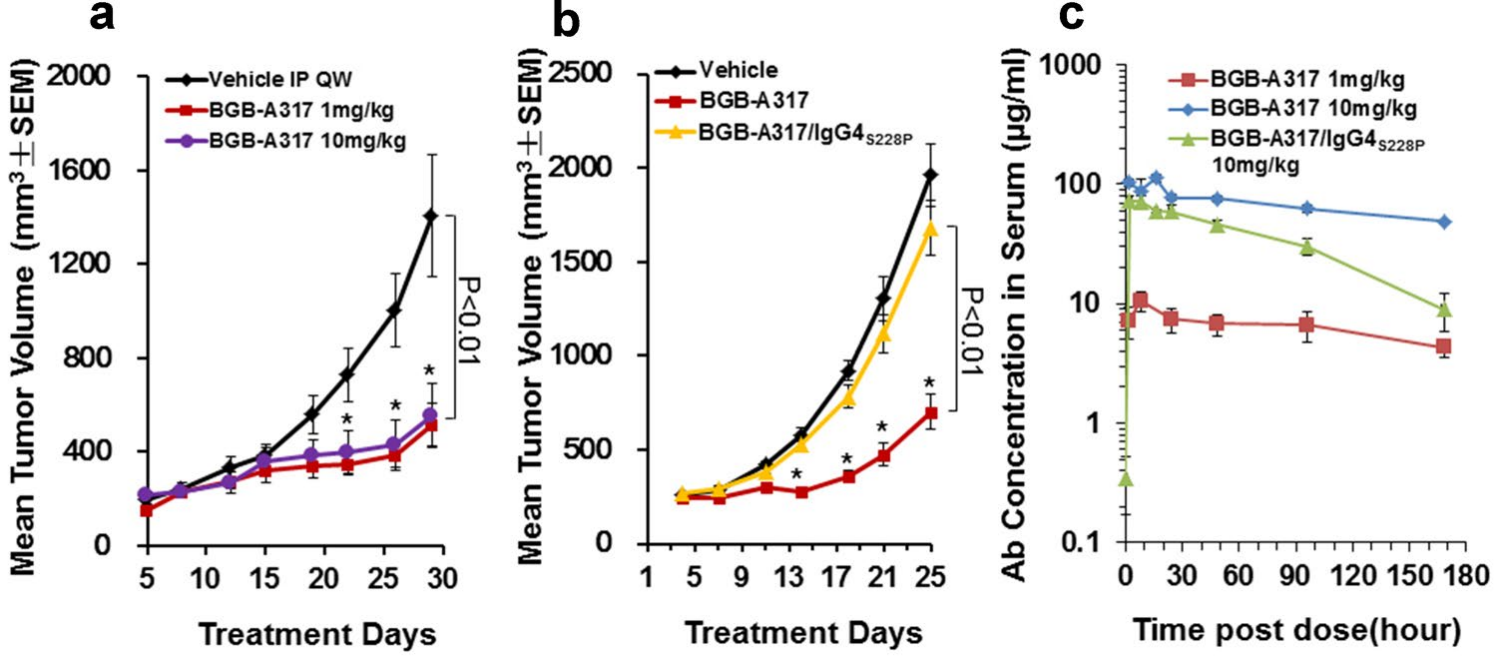
Anti-tumor activities of BGB-A317 and BGB-A317/IgG4_S228P_ in an allogenic xenograft model. **a** The anti-tumor activity of BGB-A317 at doses of 1 and 10 mg/kg, QW, i.p. was assessed in A431 allogenic xenograft model, in which PBMCs from healthy donors and A431 cancer cells were co-injected subcutaneously into NOD/SCID mice. Each treatment group had ten mice. **b** The anti-tumor activities of BGB-A317 (*n* = 11) and BGB-A317/IgG4_S228P_ (*n* = 13) were compared at the same dose of 10 mg/kg, QW and in the same model as in (**a**). **c** PK curves of BGB-A317 (1 and 10 mg/kg, *n* = 3–4) and BGB-A317/IgG4_S228P_ (10 mg/kg, *n* = 4) with a single dose treatment in NOD/SCID mice is shown. The PK parameters are summarized in Suppl. Table 2

Because BGB-A317 and BGB-A317/IgG4_S228P_ can only recognize human, but not mouse PD-1, their anti-tumor activity was tested in vivo in a xenograft allogenic cancer model, in which a human tumor cell line A431 and human PBMCs were co-injected subcutaneously into NOD/SCID mice. As shown in Fig. 4a, b, BGB-A317 exerted significant tumor growth inhibition and the dosages of 1 and 10 mg/kg did almost equally well (Fig. 4a), whereas 10 mg/kg of BGB-A317/IgG4_S228P_ had no inhibitory effect on tumor growth (Fig. 4b). Analysis of the pharmacokinetics (PK) of the two antibodies ruled out the possibility that the observed differences in their anti-tumor activities were due to the PK or lack of therapeutic antibody in sera, because BGB-A317/IgG4_S228P_ dosed at 10 mg/kg had much higher AUC and trough sera concentrations than BGB-A317 dosed at 1 mg/kg (Fig. 4c and Suppl. Table 2). Therefore, the underlying mechanism at work here appears to be related to differences in the Fc-mediated effector functions.

### BGB-A317/IgG4_S228P_ enriches murine FcγRI^+^ macrophages and reduces PD-1^+^ and CD8^+^ T cells in tumor microenvironment (TME)

**Fig. 5:**
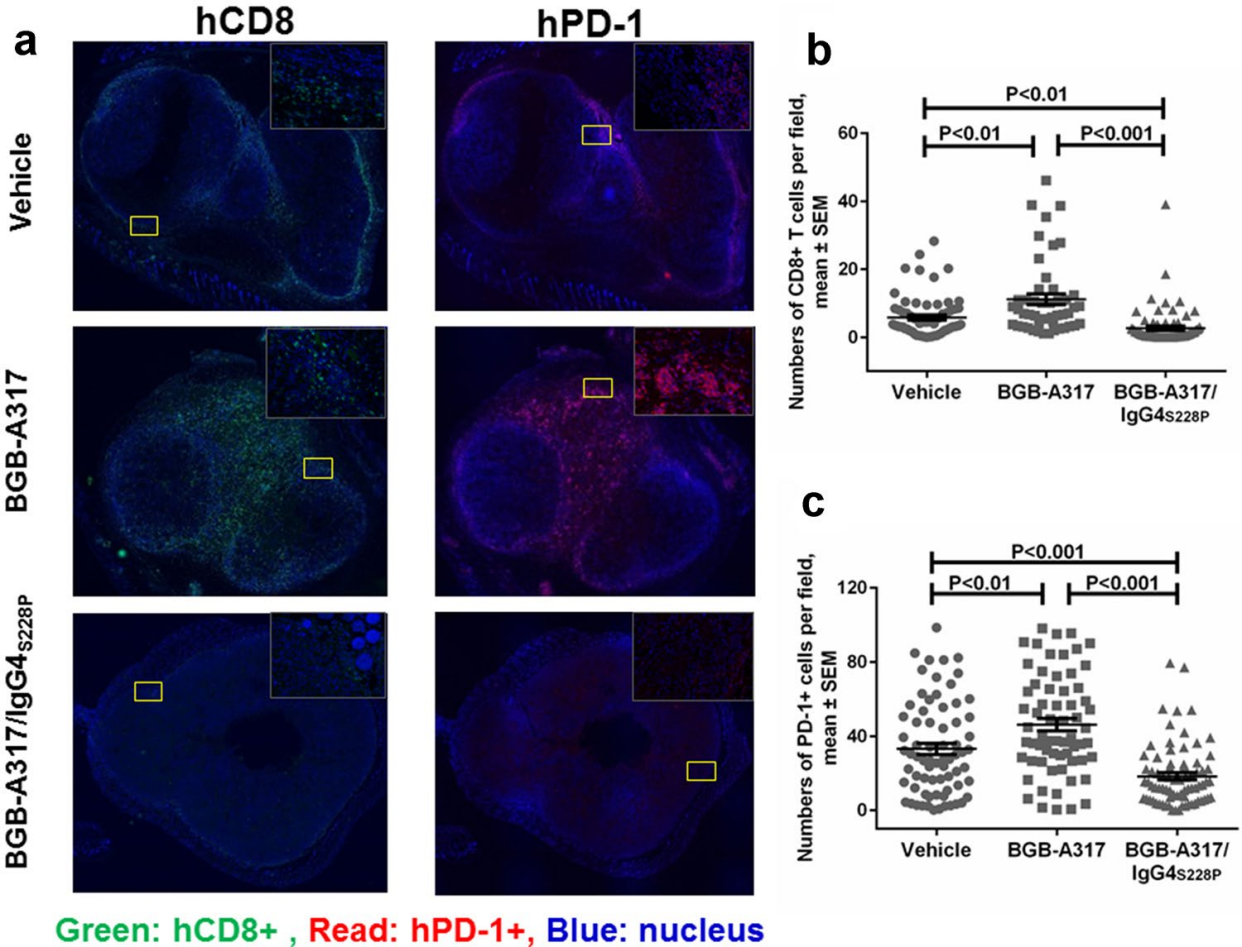
The effect of anti-PD-1 antibody treatment on tumor-infiltrating T cells. **a** Representative images of hCD8 and hPD-1 immunofluorescence staining of tumor tissues from BGB-A317- or BGB-A317/IgG4_S228P_-treated mice. **b, c** Quantified result of tumor-infiltrating hCD8 and hPD-1 positive cell numbers in each indicated group (*n* = 5). Ten images were taken for each tumor tissue sample by Vectra. The relative numbers of immunofluorescence-positive cells were quantified using inForm software

Since we observed significantly higher levels of macrophage-mediated ADCP of PD-1^+^ T cells with BGB-A317/IgG4_S228P_ than with BGB-A317 in the in vitro assay, we sought to obtain evidences that similar phenomenon might occur in TME after anti-PD-1 antibody treatment. Immunofluorescence assay showed that BGB-A317 treatment resulted in the highest density of CD8^+^ (Fig. 5a, b) and PD-1^+^ (Fig. 5a, c) T cells inside tumors. In contrast, BGB-A317/IgG4_S228P_ treatment significantly reduced the density of CD8^+^ (Fig. 5a, b) and PD-1^+^ T cells in tumors (Fig. 5a, c). IHC and analysis of fresh tumor tissues by FACS demonstrated similar findings (Suppl. Figure 4).

**Fig. 6:**
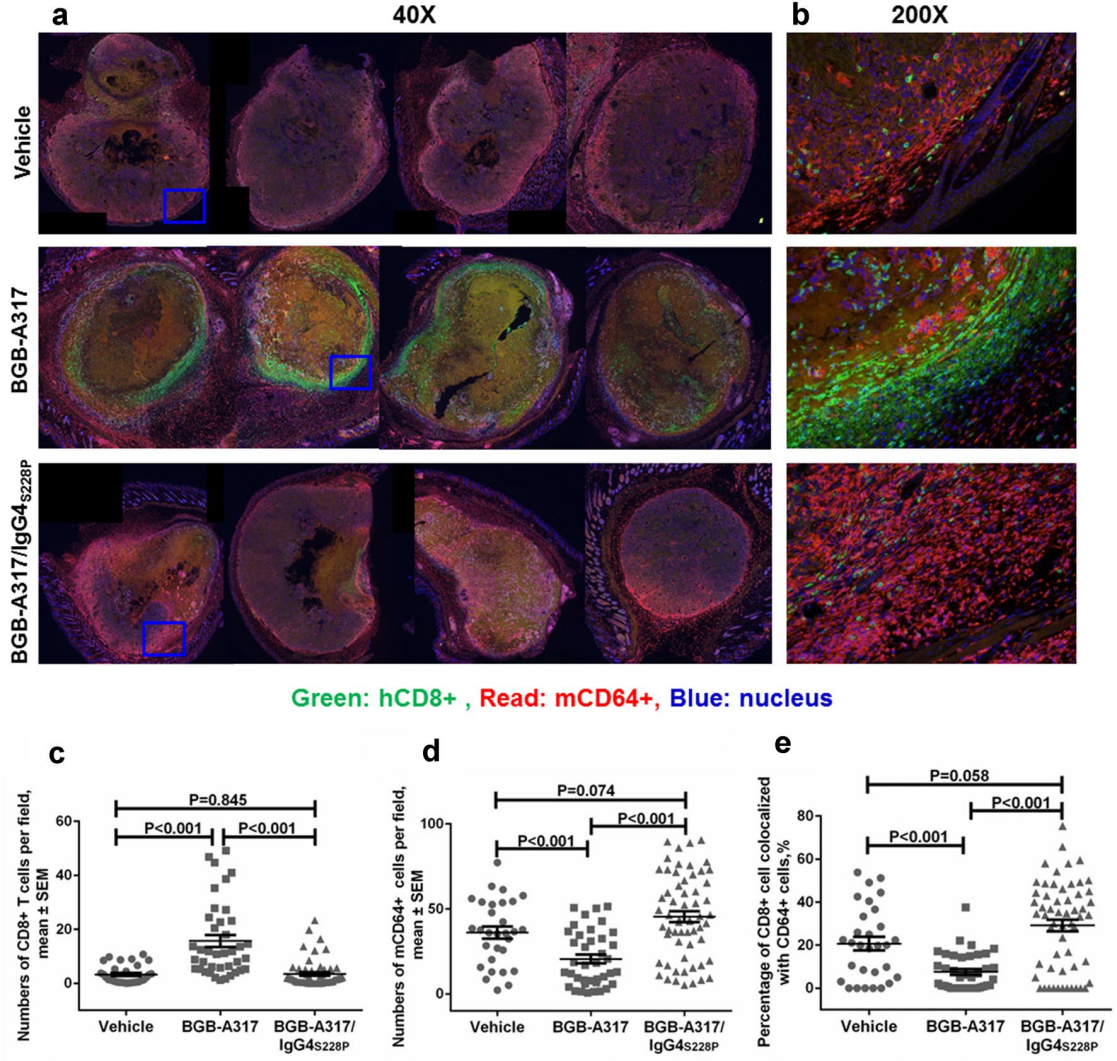
Crosslinking of T cells and FcγRI positive cells in vivo. **a** Representative low power (40×) images of multiplex immunofluorescence staining of whole mount tumors treated with BGB-A317 or BGB-A317/IgG4_S228P_. The xenograft tumor model was generated by subcutaneously implanting the allogenic human epidermoid cancer cells (A341 cell line) and PBMCs in NOD/SCID mice. Representative high power images (200×) were shown in (**b**). The biomarkers are indicated by green (hCD8), red (mCD64) and blue (nucleus), respectively. **c**–**e** Quantified results of hCD8, mCD64 staining intensity and co-localization of hCD8 and mCD64 in each indicated group (*n* = 5). Six to ten images in each tumor sample were captured by Vectra, and the staining intensity and co-localization was quantified using inForm software. (Note: CD64 = FcγRIα)

To explore the role of FcγRI (CD64) in the significant reduction of intratumoral PD-1^+^ and CD8^+^ T cells after BGB-A317/IgG4_S228P_ treatment, tumor tissues were stained for both human CD64 (hCD64) and its mouse counterpart, mCD64, which can also be bound by human IgG4 and mediate the antibody-dependent effector functions *via* mCD64^+^ cells [22]. The result showed that the density of hCD64^+^ cells was very low, whereas mCD64^+^ cells were highly enriched in TME (Suppl. Figure 2). A clear inverse correlation of higher density of mCD64^+^ cells and lower density of CD8^+^ T cells was observed in the tumors treated with BGB-A317/IgG4_S228P_ (Fig. 6a–d). On the contrary, BGB-A317-treated tumors displayed higher levels of infiltration of CD8^+^ T cells, but lower density of mCD64^+^ cells (Fig. 6a–d).

To further clarify the identity of these mCD64^+^ cells, we performed multiplex immune-fluorescent staining of the tumor tissue sections using antibodies against murine macrophage markers F4/80, CD11b [23, 24], neutrophil marker [25] and mCD64. Our results showed that the majority of tumor-infiltrating mCD64^+^ cells were stained positive with F4/80 and/or CD11b, not with the neutrophil markers, indicating that these mCD64^+^ cells are mainly murine macrophages not neutrophils (Suppl. Figure 5a). A close examination of the xenograft tumors further revealed that the hPD-1^+^ T cells and mCD64^+^ macrophages were frequently localized in contact with each other, implying a high probability of crosslinking of hPD-1 and mCD64 in the presence of BGB-A317/IgG4_S228P_ in vivo (Suppl. Figure 5b). In addition, more co-localizations of hCD8^+^ and mCD64^+^ cells were observed in BGB-A317/IgG4_S228P_ treated tumors than the ones treated with BGB-A317 (Fig. 6e). The cytotoxic T cells (CTLs) in CD8^+^ T cells was identified by the positive staining of perforin, CD107a (Lysosomal-associated membrane protein 1, LAMP1) and granzyme B (Suppl. Figure 6a–c). More CTLs were detected in BGB-A317 treated tumors than those treated with BGB-A317/IgG4_S228P_ (Suppl. Figure 6d–f).

## Discussion

In this report, we showed that the anti-PD-1 mAb with IgG4_S228P_ retained high affinity binding to FcγRI and mediated crosslinking of PD-1 and FcγRI, which brought PD-1^+^ T cells and FcγRI^+^ macrophages together and induced profound changes in intracellular signaling and biological activities, including the inhibition of PD-1^+^ T-cell functions, induction of ADCP and IL-10 secretion by macrophages. It was well documented that macrophages were present with high frequency and abundance in some cancer types [26]. The high density of macrophages in the TME promote tumor progression, metastasis and resistance to therapies [27–29] and are associated with poor prognosis [30]. In addition, a significant portion of macrophages have been found to be in close contact with T cells in many human tumors [31, 32]. Thus, the crosslinking of PD-1 and FcγRI receptors by an anti-PD-1 mAb with IgG4_S228P_ will very likely occur in TME.

PD-1 is highly expressed in T effector cells (T_effs_) [33]. Immunotherapy with anti-PD-1 antibody is thought to protect PD-1^+^ T effector cells from PD-1 ligand engagement and its inhibitory effects. Therefore, to avoid possible killing of PD-1^+^ T_effs_ through ADCC and CDC, it is desirable to have the effector functions of PD-1 mAb removed. Dahan et al. using FcγR-null mouse cancer models demonstrated that the anti-tumor efficacy of anti-PD-1 mIgG2a mAbs is significantly better in FcγR-null than in FcγR-competent mice. Human IgG4 antibodies usually do not induce significant ADCC and CDC, since IgG4 binds to FcγRIII with very low affinity and lacks binding to C1q [10, 34, 35]. However, IgG4 antibodies still retain high affinity to FcγRI as shown by our SPR assay and others [10]. In line with these findings, therapeutic IgG4 antibodies have been shown to deplete target cells in both humans and a humanized mouse model [36, 37]. Our in vitro assay showed that BGB-A317/IgG4_S228P_ induced significant ADCP than BGB-A317, raising the possibility that FcγRI binding might lead to the killing of PD-1^+^ TILs by TAMs and inferior anti-tumor activity. In fact, reduced anti-tumor efficacy was observed in IgG4_S228P_-treated groups as compared with BGB-A317 treatment in a xenograft model. Moreover, fewer numbers of CD8^+^ and PD-1^+^ T cells within tumors were seen after BGB-A317/IgG4_S228P_ treatment, which was also consistent with the presence of high density of murine CD64^+^ cells within tumors. The results of this study clearly demonstrated that anti-PD-1 antibodies with or without FcγRI-mediated effector functions exert dramatically different pharmacodynamics effects in anti-cancer activity when there is a high density of FcγRI^+^ cells (primarily macrophages) present in TME.

There is growing evidence suggesting that FcγRI signaling in macrophages may mediate anti-inflammatory effects. Previous studies have shown that ligation of FcγRI on macrophages could promote the production of the anti-inflammatory cytokine IL-10 and dampen the responses to IFN-γ [19, 38]. In addition, FcγRI is critical for TGFβ2-treated macrophage-induced tolerance and plays an important role in IgG4-induced M2 macrophage generation [20, 39]. In a comparative study on the activation of IL-10 production in macrophages, IgG4 has shown similar potency as IgG1 [40].

This study clearly showed that anti-PD-1 antibody with FcγRI-binding activity had significantly reduced anti-tumor efficacy in the NOD/SCID mouse cancer model with allogenic xenograft of cancer cells (human epidermoid cell line, A431) and human PBMCs. NOD/SCID mice are devoid of T, B cells and IgGs, but have myeloid-derived lineage cells such as macrophages [41]. Therefore, macrophage FcγRI-mediated ADCP could efficiently eliminate PD-1^+^ T cells targeted by a regular PD-1 antibody [42] (also see Fig. 5). In clinic, both nivolumab and pembrolizumab showed clinical efficacy in multiple cancer types [43]. However, the response rate is typically less than 30%. Lack of T-cell infiltration, PD-L1 expression and presence of other check-point molecules were attributed to the lack of responses in anti-PD-1 Ab therapy in most patients [43]. We previously compared the efficacy of anti-PD-1 antibodies (BGB-A317, nivolumab and pembrolizumab) using tumor samples from colorectal liver metastasis (CLM) patients [44]. BGB-A317 demonstrated better activation of TILs from CLM tumors where macrophages were more abundant. All these studies suggested that beyond the reasons described above, prevalence of FcγRI^+^ macrophages infiltration in the TME might also play negative roles in anti-PD-1 antibody (such as nivolumab and pembrolizumab) treatment. Arlauckas et al. recently showed in a mouse model that anti-PD-1 antibody could be transferred from PD-1^+^ T cells to macrophages via FcγR-dependent manner [45], supporting our findings that tumor-associated macrophages and FcγRI can negatively impact on anti-PD-1 antibody-mediated anti-tumor efficacy.

In summary, our study demonstrated that an anti-PD-1 IgG4_S228P_ antibody could mediate crosslinking between PD-1^+^ T cells and FcγRI^+^ macrophages, resulting in macrophage-mediated phagocytosis of PD-1^+^ T cells, conversion of PD-1 blockade to activating and induction of *IL-10* gene expression, and, therefore, dampening T-cell-mediated immune responses. Together, the ability to bind FcγRI by an anti-PD-1 mAb could significantly impair its anti-tumor activity, especially in the TME where macrophages are highly enriched. However, such negative impact on anti-tumor activity should be limited if the effector functions of an anti-PD-1 antibody is removed. Further clinical studies on PD-1 therapy may shed more light on the issue.

### Electronic supplementary material

Below is the link to the electronic supplementary material.

Supplementary material 1 (PDF 7628 KB)

## Abbreviations

ADCP: Antibody-dependent cell phagocytosis
CTL: Cytotoxic T cell
FcγR: Fc gamma receptor
IL-2: Interleukin 2
IL-6: Interleukin 6
IL-8: Interleukin 8
IL-10: Interleukin 10
LAMP1: Lysosomal-associated membrane protein 1
M2 macrophages: Type 2 macrophages
MDSC: Myeloid-derived suppressor cell
MFI: Mean fluorescence intensity
PBMC: Peripheral blood mononuclear cell
PD-1: Programmed cell death-1
PD-L1: Programmed cell death-ligand 1
T_eff_: T effector cell
TNF-α: Tumor necrosis factor-α
