# Supplementary material for: The binding of an anti-PD-1 antibody to FcγRΙ has a profound impact on its biological functions
Source: Cancer Immunol Immunother. 2018 Apr 23;67(7):1079–90. doi: 10.1007/s00262-018-2160-x (PMC6006217; doi:10.1007/s00262-018-2160-x)
Supplement: Supplementary file 1 — Supplementary material 1 (PDF 7628 KB) [file 262_2018_2160_MOESM1_ESM.pdf]

Supplementary tables

Supplementary table 1. Comparison of FcγRI-binding profiles by BGB-A317 and BGB-A317/$IgG4_{S228P}$

	k_a (1/Ms)	k_d (1/s)	K_A (1/M)	K_D (M)
BGB-A317/$IgG4_{S228P}$	7.12×10^{5}	1.99×10^{-3}	3.57×10^{8}	2.80×10^{-9}
BGB-A317	ND	ND	ND	ND

K_A: affinity. K_D: dissociation constant. ND: not detectable.

Supplementary table 2. Comparison of PK parameters between BGB-A317 and BGB-A317/$IgG4_{S228P}$

	Dose (mg/kg, QW)	C_{max} (μg/ml)	$AUC_{0\text{-}168h}$ (μg/ml-h)
BGB-A317	1	10.6	1087
BGB-A317	10	112.8	11396
BGB-A317/$IgG4_{S228P}$	10	73.0	6000

Supplementary figure 1

```
             1         11        21        31        41        51        61        71        81
IgG4 S228P   ASTKGPSVFPLAPCSRSTSESTAALGCLVKDYFPEPVTVSWNSGALTSGVHTFPAVLQSSGLYSLSSVVTVPSSSLGTKTYTCNVDHKPS
IgG4 Wt      ASTKGPSVFPLAPCSRSTSESTAALGCLVKDYFPEPVTVSWNSGALTSGVHTFPAVLQSSGLYSLSSVVTVPSSSLGTKTYTCNVDHKPS
BGB-A317     ASTKGPSVFPLAPCSRSTSESTAALGCLVKDYFPEPVTVSWNSGALTSGVHTFPAVLQSSGLYSLSSVVTVPSSSLGTKTYTCNVDHKPS
Consensus    astkgpsvfplapcsrstsestaalgclvkdyfpepvtvswnsgaltsgvhtfpavlqssglyslssvvtvpssslgtktytcnvdhkps

             91        101       111       121       131       141       151       161       171
IgG4 S228P   NTKVDKRVESKYGPPCPPCPAPEFLGGPSVFLFPPKPKDTLMISRTPEVTCVVVDVSQEDPEVQFNWYVDGVEVHNAKTKPREEQFNSTY
IgG4 Wt      NTKVDKRVESKYGPPCPSCPAPEFLGGPSVFLFPPKPKDTLMISRTPEVTCVVVDVSQEDPEVQFNWYVDGVEVHNAKTKPREEQFNSTY
BGB-A317     NTKVDKRVESKYGPPCPPCPAPPVAGGPSVFLFPPKPKDTLMISRTPEVTCVVVAVSQEDPEVQFNWYVDGVEVHNAKTKPREEQFNSTY
Consensus    ntkvdkrveskygppcppcpapeflggpsvflfppkpkdtlmisrtpevtcvvvdvsqedpevqfnwyvdgvevhnaktkpreeqfnsty

             181       191       201       211       221       231       241       251       261
IgG4 S228P   RVVSVLTVLHQDWLNGKEYKCKVSNKGLPSSIEKTISKAKGQPREPQVYTLPPSQEEMTKNQVSLTCLVKGFYPSDIAVEWESNGQPENN
IgG4 Wt      RVVSVLTVLHQDWLNGKEYKCKVSNKGLPSSIEKTISKAKGQPREPQVYTLPPSQEEMTKNQVSLTCLVKGFYPSDIAVEWESNGQPENN
BGB-A317     RVVSVLTVVHQDWLNGKEYKCKVSNKGLPSSIEKTISKAKGQPREPQVYTLPPSQEEMTKNQVSLTCLVKGFYPSDIAVEWESNGQPENN
Consensus    rvvsvltvlhqdwlngkeykckvsnkglpssiektiskakgqprepqvytlppsqeemtknqvsltclvkgfypsdiavewesngqpenn

             271       281       291       301       311       321       331       341       351
IgG4 S228P   YKTTPPVLDSDGSFFLYSRLTVDKSRWQEGNVFSCSVMHEALHNHYTQKSLSLSLGK
IgG4 Wt      YKTTPPVLDSDGSFFLYSRLTVDKSRWQEGNVFSCSVMHEALHNHYTQKSLSLSLGK
BGB-A317     YKTTPPVLDSDGSFFLYSKLTVDKSRWQEGNVFSCSVMHEALHNHYTQKSLSLSLGK
Consensus    ykttppvldsdgsfflysrltvdksrwqegnvfscsvmhealhnhytqkslslslgk
```

Amino acid sequence alignment of the Fc-hinge regions of IgG4$_{S228P}$, IgG4wt and BGB-A317 (or BGB-A317/IgG4variant). Differences in the sequences are highlighted in blue.

Supplementary figure 2

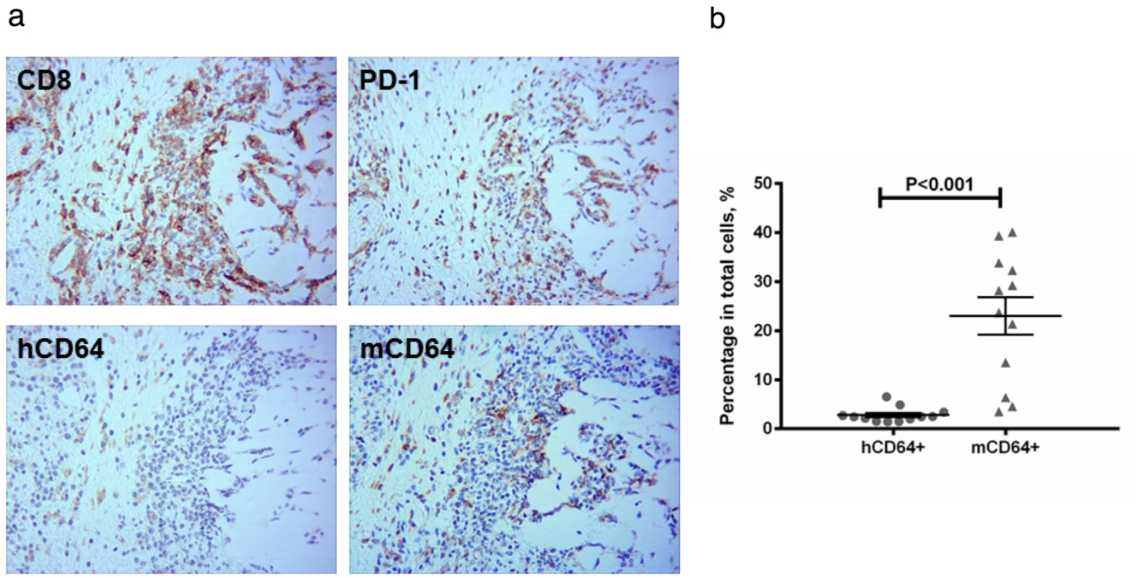

Characterization of human and mouse CD64^{+} cells within A431 allogeneic xenograft model. (**a**) Representative images of mCD64 and hCD64 IHC staining. (**b**) Comparison of mCD64^{+} and hCD64^{+} cells within tumor tissues by FACS. (Note: CD64 = FcγRIα)

Supplementary figure 3

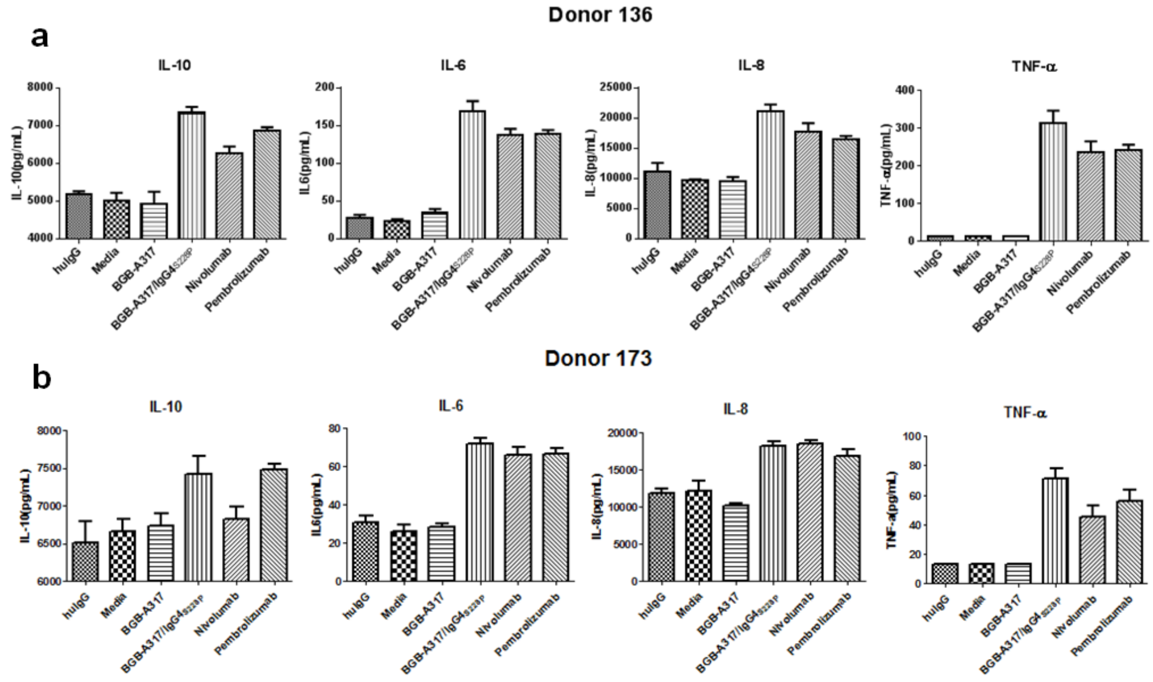

Cytokine production by M2 macrophages. To determine whether anti-PD-1 antibodies with FcγR-binding ability could activate primary M2 macrophages to increase cytokine production in response to the cross-linking with PD-1-positive HuT78/PD-1 cells, the in vitro-differentiated M2 cells (as described in the Methods and Materials) were co-cultured with HuT78/PD-1 cells in the presence of anti-PD-1 Abs at the final concentration of 10 μg/ml in 96-well v-bottomed plates. After overnight co-culture, cell-free supernatants were assayed for cytokines using a multiplex kit (Millipore, HCYTOMAG-60K) and Luminex 200 (Merck). The results of triplicate data points with M2 macrophages from two individuals (donor 136, **a** and donor 173, **b**) were presented as mean + SD (error bar).

Supplementary figure 4

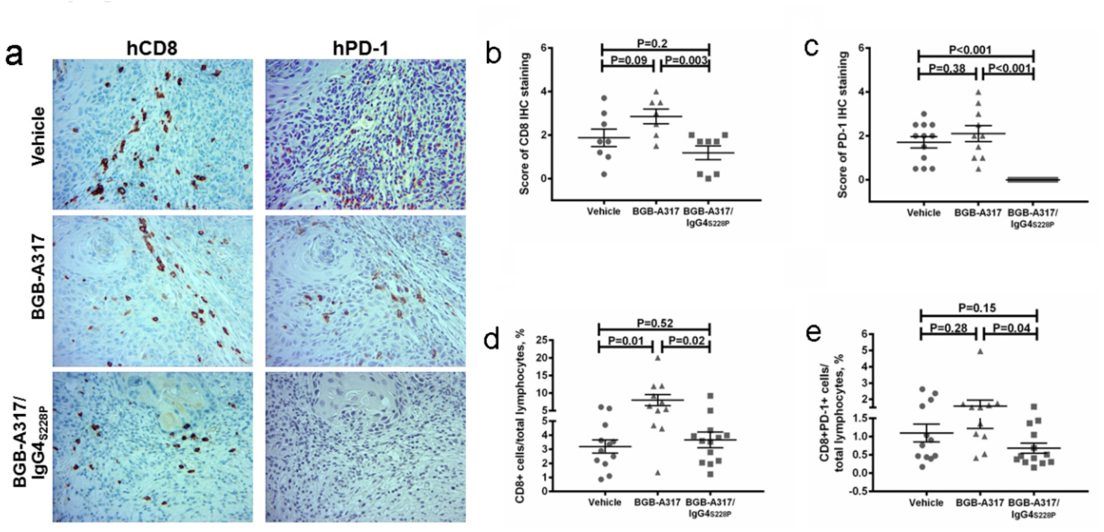

The effect of anti-PD-1 antibody treatment on tumor-infiltrating T cells. (**a**) Representative images of hCD8 and hPD-1 IHC staining on tumor tissues from BGB-A317- or BGB-A317/IgG4$_{S228P}$ treated mice. (**b**-**c**) Quantified result of hCD8 and hPD-1 IHC staining intensity in indicated groups. The IHC staining intensity was scored by 2 pathologists independently. (**d**-**e**) The result of FACS analysis of tumor infiltrated hCD8 and hPD-1 positive cells in indicated groups.

Supplementary figure 5

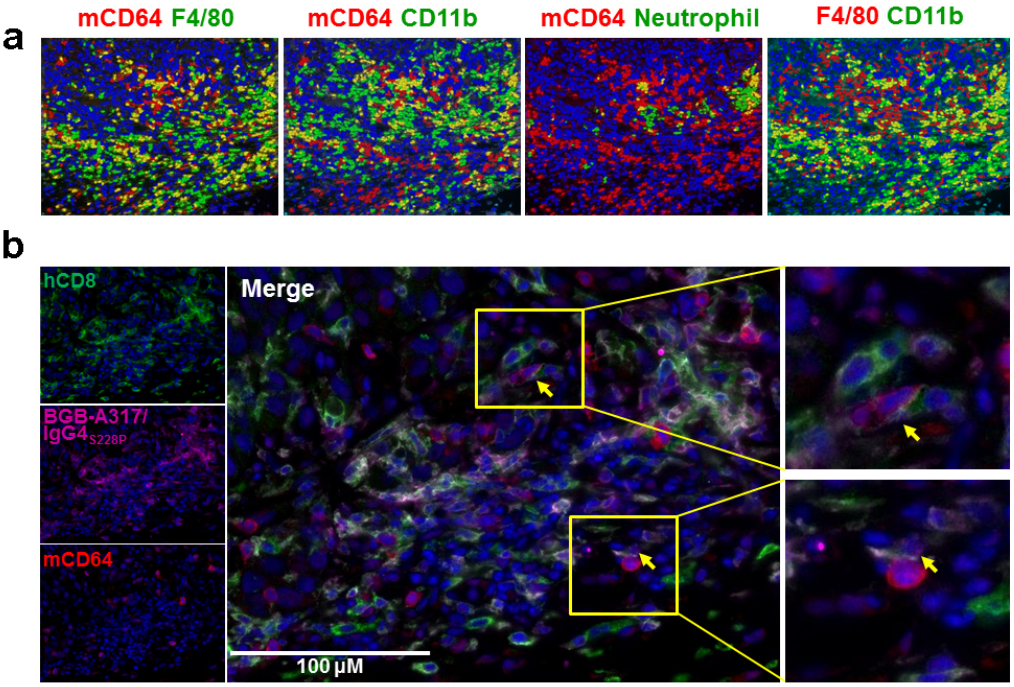

Multiplex immunofluorescence analysis of immune biomarkers in A431 tumor samples. (**a**) Digitized images of immunofluorescent staining of BGB-A317/IgG4$_{S228P}$-treated tumors. The same tumor section sample was stained with anti-mCD64, anti-F4/80, anti-murine CD11b, and neutrophil antibodies. Blue color presents staining of nuclei. Other immune cell biomarkers are coded by colored words on the top of the images. The immune staining photos were analyzed by inForm, and double positive cells are indicated in yellow. (**b**) Co-localization of BGB-A317/IgG4$_{S228P}$ with CD8^{+} T cells and mCD64^{+} immune cells. Arrows indicate the pockets of cells co-stained positively at the junction sites by the three biomarkers: hCD8, BGB-A317/IgG4$_{S228P}$ and mCD64. BGB-A317/IgG4$_{S228P}$ was biotinylated and dosed at 10 mg/kg to NOD/SCID mice co-transplanted with A431 cells and human PBMCs twice a week for 3 weeks. One day after the last dose, the mice were sacrificed and the tumor tissues were processed for immunofluorescence staining by anti-hCD8, anti-mCD64 and streptavidin Alexa Fluor® 647 conjugate (Life Technologies). The images were captured by Vectra. (Note: CD64 = FcγRIα)

Supplementary figure 6

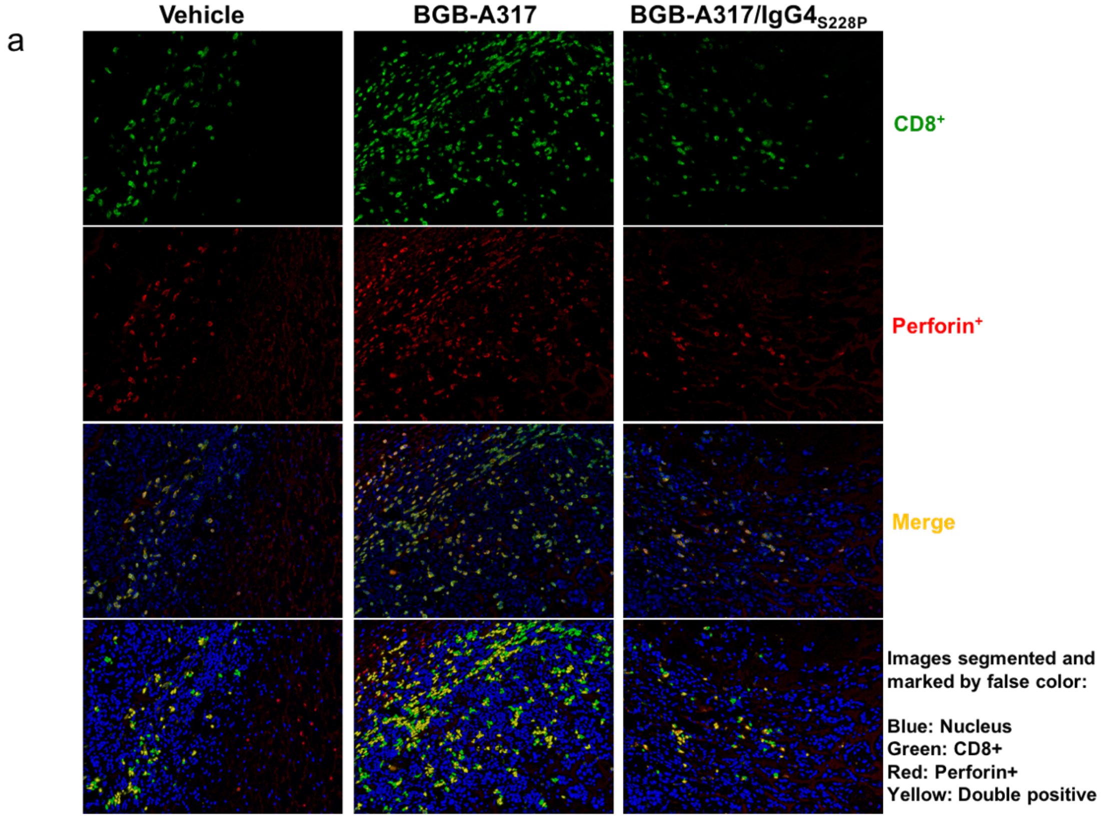

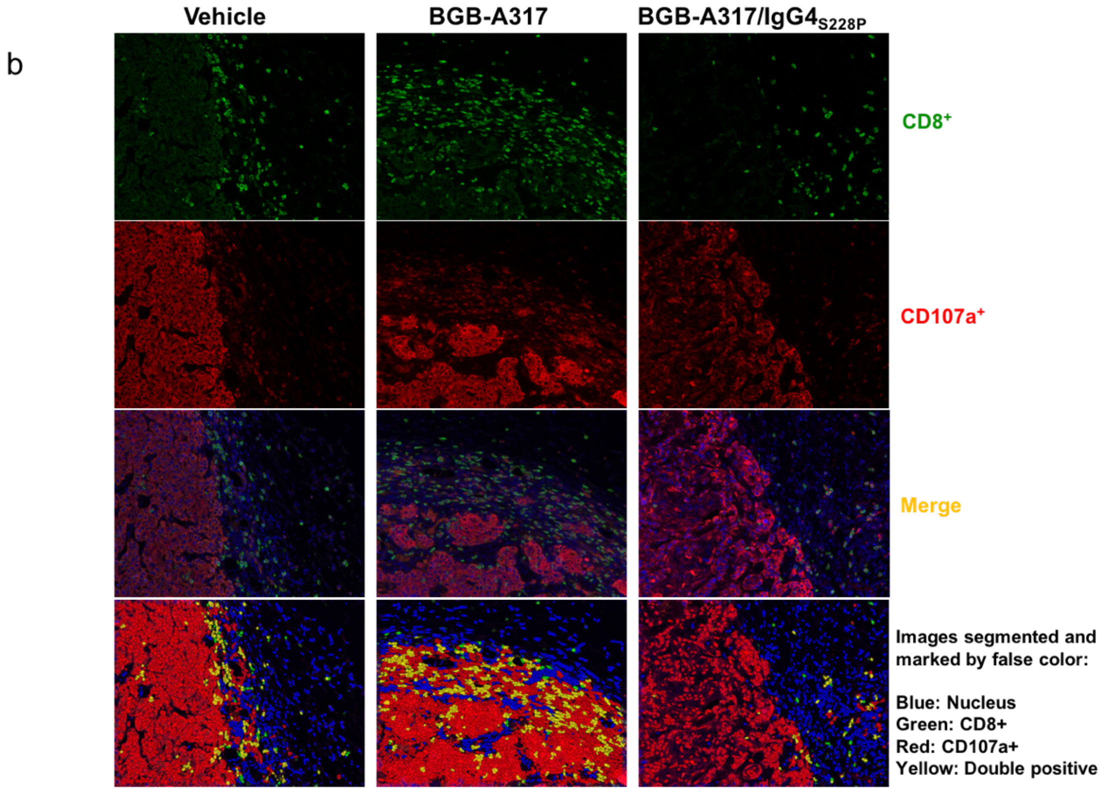

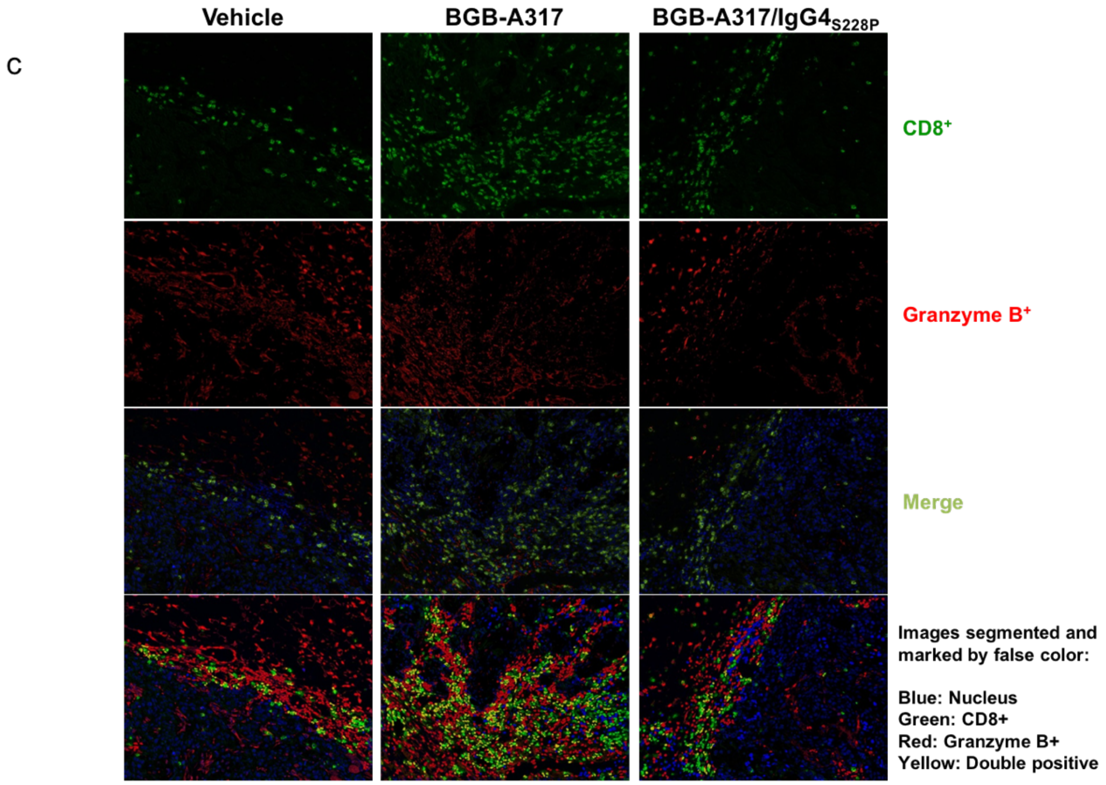

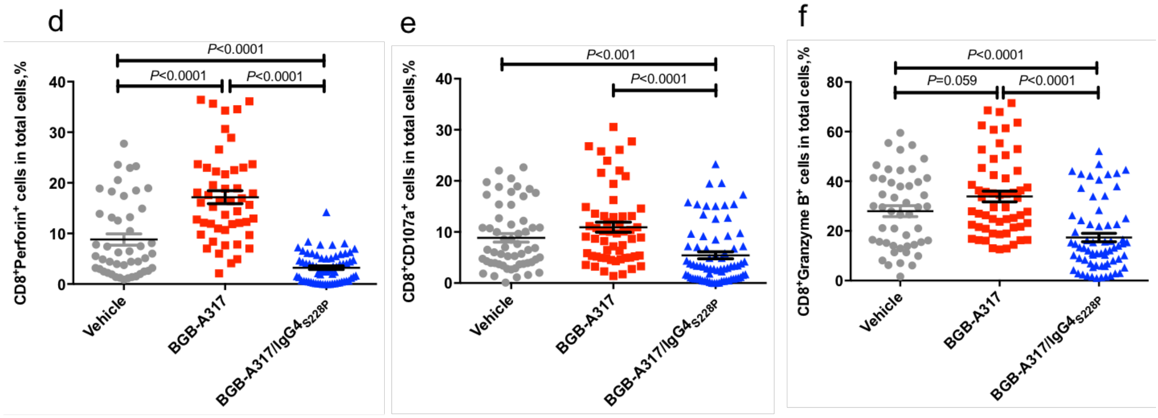

Immunofluorescence analysis of cytotoxic T cell (CTL) markers in A431 tumor samples. The tumor tissue samples of A431 xenograft mice treated with BGB-317 or BGB-A317/IgG4S228P were stained with antibodies against perforin (ab180773, Abcam), CD107a (Lysosomal-associated membrane protein 1, LAMP1, 11215-H08H, Sinobiological), or granzyme B (10345-H08H, Sinobiological) in together with CD8 (SP57, Ventana), respectively. **(a-c)** The representative images of the colocalization of perforin, CD107a or granzyme B with $CD8^+$ T cells. The immune cell biomarkers are

color-coded by the words on the right of the images. Blue color presents staining of nuclei. The staining of perforin and granzyme B was only observed on non-tumor cells, while CD107a was also detected on tumor cells. (**d-f**) Quantification of the percentage of perforin^{+}/CD8^{+}, CD107a^{+}/CD8^{+} and granzyme B^{+}/CD8^{+} T cells within total cells in tumor samples treated with BGB-A317 or BGB-A317/IgG4S228P. The horizontal bars and P values indicates the statistical significances between the treatment groups. The images were captured by Vectra and analyzed by inForm.
